# Supplementary material for: Experiences and impact of chronic pain in South Africans living in a rural area: a qualitative study
Source: BMJ Open. 2025 Dec 24;15(12):e103307. doi: 10.1136/bmjopen-2025-103307 (PMC12742062; doi:10.1136/bmjopen-2025-103307)
Supplement: online supplemental file 1 [file bmjopen-15-12-s001.docx]

**Ndlovu study**

**Interview guide for in-depth interview**

The following interview guide, drawn from grounded theory, will guide the interview:

Different sections will be asked depending on whether the individual has HIV and/or pain.

***For all participants:***

1. How long have you been living in this area?
2. What is it like to live in this community?
   1. What are the jobs like around here?
      1. How easy or difficult is it to find a job here?
   2. Tell me about your friends here?
      1. What do you talk about with your friends?
   3. How do people help when things get difficult?
3. Tell me what you think about the health care system here.
   1. E.g. What do you think about the service you receive at the clinic/hospital, the nurses, doctors and other service providers? (please ask for each)
4. Could you describe a typical day for you?
   1. What would you do on a normal day? How busy is it?

**For those living with HIV**

1. Would you tell me about living with HIV?
   1. When were you diagnosed with HIV?
   2. How has life been since then?
   3. How does living with HIV affect you?

(E.g. emotionally, physically, work, study, socially, sexually/in relationships) (ask for each separately)

- 1. How does living with HIV stop you from doing anything?

(E.g. emotionally, physically, work, study, socially, sexually/in relationships) (ask for each separately)

1. Have you told your family/spouse/partner/friends/priest about having HIV? (Please ask for each separately)
   1. If yes, for the people you told you have HIV, how did they react?
   2. For the people you told, why did you tell them?
   3. If no, can you tell me why you didn’t tell them?
2. Can you ask for help if you need it, either at work, in the community or from friends or family? Is there support available?
   1. What kind?

***For those with pain***

1. Tell me about your pain?
   1. When did it start?
   2. What does it feel like?
      1. How would you describe it?
      2. How often do you feel the pain?
2. Why do you think you have pain?/What do you think is the cause of your pain?
3. What do you do to relieve the pain? e.g. medications, activities/rest.
4. When the pain is bad:
   1. What are you still able to do?
   2. What activities can’t you do?
   3. Why can’t you do those activities?
      1. Would it make your pain worse? Would you be unable to complete the task?

Tell me about a time you were able to ask for help when the pain was bad.

1. Have you told your family/spouse/partner/friends/priest about your pain?
   1. For those people you told, did you tell them just that you had pain, or actually how much pain you have?
   2. What were your reasons for telling them about your pain?
   3. For those people you told how did they react when you told them about your pain?
      1. Can you tell me about a time your shared living with pain with someone?
      2. How did it feel to tell them?
   4. If no, can you tell me why you didn’t tell them how much pain you were in?
      1. How did you feel not being able to tell them, either at all or not the full extent of your pain?
2. Have you told a doctor or nurse about your pain? (please ask about the doctor first and then ask about the nurse)
   1. May you please tell me how that went?
   2. Were you able to tell them how much pain you had or just that you had pain?
      1. How do think they were able to help?
3. If no, you weren’t able to tell a doctor or nurse, can you tell me why you didn’t tell them or why you didn’t tell them how much pain you were in? (please ask about the doctor first and then ask about the nurse)

**For those living with HIV *and* pain**

1. What is it like to live with both HIV and pain?
2. Is there support available, either at work, in the community or from friends or family? Can you ask for help?
   1. What kind of support is there?
   2. Do you know of other people who live with both HIV and pain?
